# Students’ awareness of malaria at the beginning of national malaria elimination programme in China

**DOI:** 10.1186/1475-2875-12-237

**Published:** 2013-07-12

**Authors:** Jian-hai Yin, Ru-bo Wang, Zhi-gui Xia, Shui-sen Zhou, Xiao-nong Zhou, Qing-feng Zhang, Xin-yu Feng

**Affiliations:** 1 National Institute of Parasitic Diseases, Chinese Center for Disease Control and Prevention, Shanghai 200025, People’s Republic of China; 2 WHO Collaborating Centre for Malaria, Schistosomiasis and Filariasis, Shanghai 200025, People’s Republic of China; 3 Key Laboratory of Parasite and Vector Biology, Ministry of Health, Shanghai 200025, People’s Republic of China

**Keywords:** Awareness, Malaria, Students, China

## Abstract

**Background:**

In the battle against malaria in China, the rate of elementary and high school students’ awareness on malaria knowledge is an important index for malaria elimination, but only rare data is available. This study aimed to investigate the level of malaria awareness in students at elementary and high schools in malaria endemic areas of China, and to provide the baseline information for the malaria elimination.

**Methods:**

This cross-sectional survey was conducted in 20 different malaria-endemic provinces in the first year of China’s National Malaria Elimination Programme (NMEP). A structured questionnaire was administrated to students at elementary and high schools enrolled. A total of 44,519 questionnaires were effective while 1,220 were excluded because of incomplete survey responses.

**Results:**

More than 60% of students were aware of malaria, but only 9,013 of them answered correctly to all five questions, and there were still 1,862 students unaware of malaria. There were significant differences of the awareness of malaria among different age groups, between male and female, between two different education levels.

**Discussion:**

The study reveals that students at elementary and high school levels did not have adequate knowledge of malaria about biology, pathogenicity, transmitting vectors and preventive methods and so on at the beginning of NMEP in China. Further emphasis should be paid on health education campaigns in China to increase students’ public awareness of malaria about vector control, treatment, prevention.

## Background

Malaria, one of the category B notifiables diseases in China, was reduced dramatically as a result of unprecedented governmental efforts. In order to better protect and promote public health, to achieve the health-related Millennium Development Goals, the Chinese government launched a national campaign with the goal of eliminating the disease nationwide by 2020. An action plan of malaria elimination as a guidance document was issued in 2010 [1]. In the battle against malaria, in addition to case finding, treatment, surveillance and vector control, health education was an indispensable part [2], and the rate of elementary and high school students’ awareness on malaria knowledge will be assessed according to this action plan. It targeted that 75% of students in these two-level schools should have knowledge of malaria prevention and treatment by 2012, and 85% should reach that by 2015. Finally by 2020, the awareness of malaria should be improved further and people should participate in malaria prevention, control and elimination more proactively [1].

However, malaria awareness campaigns are often restricted to providing information at health posts, and not readily available to students at primary and high school levels. These campaigns are also usually aimed at informing people about vector control activities, and to motivate people to make use of the provided control measures such as spraying insecticide indoors, with little focus on actions that individual community members can take to not only help themselves, but to help the overall effectiveness of the local malaria control interventions [3]. It is noted that students can be important agents for change. Health education through schools can help promote a community-wide understanding of malaria and the need for control and can create a demand for health services to provide universal access to affordable and appropriate treatment [4,5].

In this study, a cross-sectional investigation into students’ perceptions of malaria-related basic knowledge at the primary and high school levels was carried out nationwide at the beginning of the NMEP, to understand the current status of students’ awareness of malaria and provide the most recent baseline data for malaria prevention and control, so that corresponding measures can be carried out, and effectiveness evaluation can be done when malaria elimination is achieved.

## Methods

### Study populations and sampling

This cross-sectional study was conducted between September and December 2010. The study population was obtained from students at elementary and high schools from malaria-endemic counties in 20 provinces in China using multistage cluster sampling. Firstly, 16 Type I malaria-endemic counties (local infections detected in three consecutive years and the annual incidences no less than 1/10,000 respectively) [1] and 78 Type II counties (local infections detected in three consecutive years and the annual incidences lower than 1/10,000 at least in one year) [1] in 20 provinces were randomly selected. Secondly, three towns representing high, middle and low malaria epidemics respectively in recent years were selected in every county. Thirdly, one elementary school and one high school were selected in each town. Finally, no fewer than 60 students in every school were surveyed.

### Questionnaire design

Demographic information comprising of code, province, city, county, school, class grade, and the individual information of name, gender and age were included in the questionnaire. For ease of understanding and operation, five simple but important malaria knowledge choice questions related to malaria vectors, main symptoms, fatal malaria species, what should do when get malaria, and malaria prevention approaches, were set to assess the perception of malaria. Each question was awarded one mark for correct answer, while each incorrect answer was given zero marks. The total marks of five questions were evaluated as follows: scores of five were considered “excellent”, while scores of three to four, one to two and zero were considered “good”, “poor” and “very poor”, respectively. And the ranks of “excellent” and “good” were considered as “aware”, otherwise were “not aware”.

### Data collection and quality control

Before the survey, investigators from provincial Center for Disease Control and Prevention (CDC)were uniformly trained by national professionals, then investigators at county level were trained by the provincials. The questionnaire was administrated to every student for fulfilment with support from professionals and schoolteachers during the first implementation year of the NMEP. All data were rechecked step-by-step and typed into a designed database.

### Data analysis

Using Microsoft Excel 2010 software, the constituent ratios of different aspects were calculated. Differences in distribution were evaluated using the chi-square (χ^2^) test by SPSS version 16.0 and *P* <0.05 was considered statistically significant.

### Ethical considerations

The study was reviewed and approved by the Ethical Committee of NIPD, China CDC. All participants included were provided verbal informed consent before admission into the study with the help of schoolteachers.

## Results

### Demographic characteristics of enrolled students

A total of 44,519 questionnaires were effective while 1,220 were excluded because of incomplete survey responses in this cross-sectional study. The majority (76.94%) of students were aged from ten to 14 years old. The ratio of male to female was close to 1. Students from elementary schools (52.22%) were slightly more than students from high schools (47.78%). The details from these questionnaires are presented in Table 1.

**Table 1:** Demographic characteristics of students of effective questionnaires (n = 44,519)

**Characteristic**	**n**	**%**
**Age group, years**		
6-9	2,446	5.49
10-14	34,254	76.94
≥15	7,819	17.56
**Sex**		
Male	22,649	50.87
Female	21,870	49.13
**Education**		
Elementary school	23,249	52.22
High school	21,270	47.78

### Knowledge of malaria

All students’ understanding of malaria knowledge was not the same. Among 44,519 effective questionnaires, the percentage of correct responses to three questions about malaria vectors, symptoms, fatal species was between 56 and 60%, and 88.09% of students had knowledge of approaches to treat malaria. However, only 35.07% of all students knew the preventive measures against malaria. The details are presented in Table 2.

**Table 2:** Distribution of knowledge of malaria related in students at elementary and high school levels in China (n = 44,519)

**Items**	**n**	**%**
**Which transmit malaria?**		
Correct	26,325	59.13
Incorrect	18,194	40.87
**Major symptoms of malaria attack?**		
Correct	26,550	59.64
Incorrect	17,969	40.36
**If left untreated, which species of malaria is fatal?**		
Correct	25,079	56.33
Incorrect	19,440	43.67
**What should be done while malaria suffered?**		
Correct	39,218	88.09
Incorrect	5,301	11.91
**What measures could prevent malaria?**		
Correct	15,615	35.07
Incorrect	28,904	64.93

### Awareness of malaria

Among the effective questionnaires, only 9,013 students responded correctly to all five questions, and 1,862 students were unaware of malaria. Only 60.46% of students were aware of malaria; most were ranked as “good” grade. Furthermore, there were significant differences of awareness of malaria among three age groups (*P* = 0.027), the percentages were 58.95% (age six to nine), 60.29% (age ten to 14) and 61.63% (age ≥15), respectively. Awareness of malaria was better in females than males (*P* = 0.022). In addition, awareness of malaria in high school students was better than in elementary school students (*P* < 0.001). The details are presented in Table 3.

**Table 3:** Characteristics of awareness of malaria in students in China

**Characteristics**	**Aware (n = 26,914)**		**Not-aware (n = 17,605)**	
	**Excellent (n = 9,013)**	**Good (n = 17,901)**	**Poor (n = 15,743)**	**Very poor (n = 1,862)**
**Age group, years**				
6-9	483	959	894	110
10-14	7,168	13,485	12,127	1,474
≥15	1,362	3,457	2,722	278
**Sex**				
Male	4,528	9,046	8,038	1,037
Female	4,485	8,855	7,705	825
**Education**				
Elementary school	4,325	9,034	8,606	1,284
High school	4,688	8,867	7,137	578

## Discussion

In China, malaria has a recorded history of more than 4,000 years and was identified as one of the top five parasitic diseases that seriously affected social-economic development. The estimated number of 1,829 malaria-endemic counties (over 70% of total counties), and more than 30 million cases with about 1% fatality rate, existed at the establishment of the People’s Republic of China in 1949. Endemic malaria was mainly caused by *Plasmodium falciparum* and *Plasmodium vivax.* Reported cases have significantly declined as endemic areas have proportionally reduced [6,7]. However, the epidemic situation of malaria in China is unstable, with long-term epidemics in the border areas of Yunnan province and vivax malaria re-emergence in central China [8,9]. In order to further reduce the malaria burden as well as respond to the global goal of malaria eradication, China launched its NMEP in 2010, with a goal of malaria elimination nationwide by 2020 [1].

Public awareness of diseases, including malaria, plays an important role in disease control and prevention, a lack of reasonable knowledge of disease leads to low detection rate, the interruption of treatment and discrimination, etc. [5,10-12]. This study was a specific, population-based, cross-sectional survey, with the aim of assessing whether students at elementary and high school levels had knowledge of malaria. One of the limitations of this study was the selection bias without regard of financial situation, local education development and family history of malaria.

Some local studies about awareness of malaria have been carried out among the populations in China, but most were focused on adults, and only a few were conducted in schools [13-15]. As well as the general public, students at elementary and high schools had a lower awareness of malaria than the findings of the present study; meanwhile, significant differences of awareness of malaria between age groups, gender and education level respectively, were found in other studies, as well as the present survey [13-15].

The results of the present study demonstrated that the level of malaria awareness among students at elementary and high schools in China was generally low at the beginning of NMEP, and malaria-related student education should be further planned and strengthened in a localized and diversified manner. Moreover, not only basic knowledge but also information such as care-seeking practices should be included into future health education campaigns on malaria [16-18]. It is suggested that malaria elimination health education should cooperate with educational sectors to improve students’ awareness of malaria. Future NMEP evaluation could use these results as a baseline for assessing the effectiveness of a programme.

## Competing interests

The authors declared that they have no competing interests. The funders had no role in study design, data collection and analysis, decision to publish, or preparation of the manuscript.

## Authors’ contributions

JHY and ZGX conceived the study. RBW and ZGX participated in questionnaire design. RBW, QFZ and XYF participated in data collection. SSZ and XNZ provided the administrative coordination. JHY analysed the data and drafted the manuscript. ZGX revised the manuscript. All authors read and approved the final manuscript.
